# Ballroom dancers exhibit a dispositional need for arousal and elevated cerebral cortical activity during preferred melodic recall

**DOI:** 10.7717/peerj.10658

**Published:** 2021-01-13

**Authors:** Xinhong Jin, Yingzhi Lu, Bradley D. Hatfield, Xiaoyu Wang, Biye Wang, Chenglin Zhou

**Affiliations:** 1 School of Psychology, Shanghai University of Sport, Shanghai, China; 2 Department of Kinesiology, University of Maryland, College Park, MD, USA; 3 Neuroscience and Cognitive Science Program, University of Maryland, College Park, MD, USA; 4 School of Biomedical Engineering, Dalian University of Technology, Dalian, China; 5 Department of Physical Education, Yangzhou University, Yangzhou, China

**Keywords:** Tempo preference, Temperament, Beta power, Dance, EEG

## Abstract

**Background:**

Although the association of human temperament and preference has been studied previously, few investigations have examined cerebral cortical activation to assess brain dynamics associated with the motivation to engage in performance. The present study adopted a personality and cognitive neuroscience approach to investigate if participation in ballroom dancing is associated with sensation-seeking temperament and elevated cerebral cortical arousal during freely chosen musical recall.

**Methods:**

Preferred tempo, indicated by tapping speed during melodic recall, and a measure of fundamental disposition or temperament were assessed in 70 ballroom dancers and 71 nondancers. All participants completed a trait personality inventory (i.e., the Chen Huichang 60 Temperaments Inventory) to determine four primary types: choleric, sanguine, phlegmatic and melancholic. Participants separately recalled their favorite musical piece and tapped to it with their index finger for 40 beats using a computer keyboard. A subset of 59 participants (29 ballroom dancers and 30 nondancers) also repeated the same tapping task while electroencephalographic (EEG) activity was recorded.

**Results:**

The results revealed that the dancers were more extraverted, indicative of a heightened need for arousal, exhibited a preference for faster musical tempo, and exhibited elevated EEG beta power during the musical recall task relative to nondancers. Paradoxically, dancers also showed elevated introversion (i.e., melancholic score) relative to nondancers, which can be resolved by consideration of interactional personality theory if one assumes reasonably that dance performance environment is perceived in a stimulating manner.

**Conclusion:**

The results are generally consistent with arousal theory, and suggest that ballroom dancers seek elevated stimulation and, thereby, choose to engage with active and energetic rhythmic auditory stimulation, thus providing the nervous system with the requisite stimulation for desired arousal. These results also suggest an underlying predisposition for engagement in ballroom dance and support the gravitational hypothesis, which propose that personality traits and perception lead to the motivation to engage in specific forms of human performance.

## Introduction

Many explanations of human behavior and performance consider an individual’s temperament or personality, which implies that there are pre-existing differences that draw people into different activities (the gravitational hypothesis, Morgan et al., 1988). Temperament depicts the initial state of personality development and relates individual differences in behavior to underlying neural networks (Rothbart, 2007), of which a fundamental element is that of activation or the need for arousal. Conceptually, temperament and experience together develop a personality, a term to qualify individual differences, although recent researches suggesting that “basic” trait of temperament and personality are conceptually related (Pauw & Mervielde, 2010; Rettew & McKee, 2005; Rothbart, 2007). The gravitational hypothesis states that persons with different personality traits engage in different types of activities, which they find most satisfying and consistent with their cognitive outlook or perception (Bird, 1979). It also proposes that people will self-select into environments most matched to their dispositions (Rubenstein et al., 2019). Based on the gravitational hypothesis, individuals characterized by specific temperament gravitate toward activities that promote a “fit” between specific personality traits and their perception of the environment (Bird, 1979; Morgan et al., 1988). In addition, according to Eysenck’s theory (Eysenck & Eysenck, 1985; Razoumnikova, 2003), individuals tend to choose activities that stimulate the nervous system to achieve their optimal or preferred level of arousal. Extroversion-introversion is one potentially important corollary of Eysenck’s theory, for example, extraverts, relative to introverts, exhibit a lower level of spontaneous cortical arousal and tend to seek more intense types of sensory stimulation to raise arousal to a desired level. Introverts, however, exhibit relatively higher chronic arousal and tend to choose activities that are less stimulating to manage their arousal (Delsing et al., 2008; Geen, 1984; Kantor-martynuska, 2009). In addition, introverts are more aroused than extraverts when engaged in activities of the same intensity, regardless of whether the sensory stimulation was preferred by extraverts or by introverts (Geen, 1984). Such findings lead to the assumption that people with different personalities seek specific levels or types of stimulation to achieve a desired and comfortable level of neural activation during performance. The focus of the present study is on the role of temperament traits in the motivation to engage in ballroom dance, which is a specific expression of human performance defined by rhythmic sensorimotor behavior.

Given that extraverts and introverts seek different levels of stimulation for achieving their desired level of neural arousal, they reasonably prefer different musical tempos. Research has revealed that the preferred tempo of movement, that is, the speed at which a motor action is naturally performed in humans, is just above 120 beats per minute (bpm) (Moelants, 2002). The preference of approximately 120 bpm in humans has also been observed for repetitive natural movements (e.g., tapping, waving, clapping, or walking) and is also found in the beat distribution of most types of music (Moelants, 2003). However, the rhythmic tempo of dance music is typically higher than that associated with the generally preferred tempo (Moelants, 2003). A long-term study that examined the distribution of dance music tempi revealed that the main peak in dance music tempo is typically located at 128 bpm, with a range of 125–130 bpm, indicative of a preferred tempo for dancing movements (i.e., approximately 128 bpm) that is higher than that for most movement-related activities (Moelants, 2008). Another study reported in the literature supported the general distribution of tempi preference around 130 bpm, which, again, is substantially higher than the naturally preferred tempo, thus explaining, in part, the need that humans feel to engage in “excited” movement while listening to dance music (Moelants, 2003). The question follows whether dancers prefer and choose such stimulating musical environments based on temperament or dispositional characteristics.

When music starts, the spontaneous movements of humans will typically synchronize with the auditory stimulation, resulting in a link between tempo and motor behavior. In addition, neural activity is positively associated with the tempo. For example, when a listener is presented with music at a tempo of 2.4 Hz, a corresponding (i.e., 2.4-Hz) neural oscillation is present in the electroencephalographic (EEG) activity (Nozaradan et al., 2011). Additional evidence has revealed endogenous cerebral cortical entrainment to auditory frequencies of 1–5 Hz, with maximum entrainment at 2 Hz (that is, 120 bpm), consistent with the intrinsic preferred natural movement tempo in humans (Moelants, 2002; Repp & Su, 2013; Will & Berg, 2007). In this manner, rhythmic body movements are also believed to be related to the preferred musical tempo (Bauer, Kreutz & Herrmann, 2015; Phillips-Silver & Trainor, 2007; Todd, Cousins & Lee, 2007). Trained dancers (i.e., those with extensive experience in the activity) are able to synchronize their body movements to every beat to create an appropriate performance. Based on the resonance theory (Large & Kolen, 1994; Large, 2008; Van Noorden & Moelants, 1999), perception of music is associated with the frequency of neural oscillation, and, accordingly, the neural oscillation is consistent with the rhythm of music.

Furthermore, a number of researchers have investigated the relationship between personality and music preference (Brown, 2012; Chamorro-Premuzic, Fagan & Furnham, 2010; Delsing et al., 2008; Greenberg et al., 2016; Rentfrow & Gosling, 2003). The type of music that people choose may serve a fundamental need and demonstrate their likes and dislikes (Colley, 2008). In this manner, music preference is associated with an individual’s personality (Schäfer & Mehlhorn, 2017) and, in regard to the arousal dimension, the personally preferred level of stimulation (Boer et al., 2011). However, music preferences are affected by much larger dimensions (types, language, etc.) that may make for difficult comparisons. The present study defined music preference by the preferred musical tempo freely recalled by participants. Although personality is characterized by multiple dimensions, only four types of temperament, fundamentally related to arousal, have been defined since the time of Hippocrates (460–370 BC). Aspects of temperament can be defined as individual differences not only in personality, but emotional, motor, and attentional reactivity, which are based and linked to an individual’s genetic endowment (Posner, Rothbart & Sheese, 2007; Rothbart, 2007; Rothbart & Derryberry, 1981). Although the study of temperament has a long history, it appears that the associations with temperament and neural activity have been examined only recently. To explore the role of temperament in trained Chinese dancers, the present study employed the widely-used and native four-type temperament scale (Chen & Liu, 2006; Li, Xi & Niu, 2011; Liu & Li, 2018; Xie & Wang, 2015) the Chen Huichang 60 Temperaments Inventory (Burger & Chen, 2000; Chen, 1995). The four types are *choleric*, which corresponds to neurotic extraverts; *sanguine*, corresponding to stable extraverts; *melancholic*, consistent with neurotic introverts; and *phlegmatic*, consistent with stable introverts (Hepburn & Eysenck, 1989). Individuals with choleric or sanguine temperaments are sociable and outgoing (i.e., extraverted), whereas those with melancholic or phlegmatic traits are more reserved and shy (i.e., introverted). Such dispositional tendencies are influential on participation in activities that are relevant to those temperamental characteristics (i.e., gravitation) in addition to any influence of social learning (i.e., reinforcement and punishment of behavior, Armstrong & Anthoney, 2009; Mckelvie, Lemieux & Stout, 2003; Rubenstein et al., 2019).

It appears that individuals perform body movements best when those movements are synchronized with their preferred tempo (Repp & Su, 2013), and the preferred tempo is believed to be associated with rhythmic body movements as well as with motor cortex activity (Bauer, Kreutz & Herrmann, 2015; Chen, Penhune & Zatorre, 2008; Grahn & Brett, 2007; Janata, Tomic & Haberman, 2012). As such, EEG offers promise to investigate the role of neural oscillations and brain dynamics in music preference and dance behavior. Regarding rhythm perception, different frequency ranges of cortical activity have been associated with different cognitive processes. Neural activation, indexed by power in the EEG beta band (13–30 Hz) is prominently associated with movement and has been observed in sensory and motor cortices (Fujioka et al., 2009; Hari & Salmelin, 1997). Bauer, Kreutz & Herrmann (2015) reported an association of between the frequency of beta activity in the motor cortex with preferred tempo, suggesting interindividual variation in tempo preference and neural activity. Importantly, a relationship was also observed between beta power and temperament. For example, Razumnikova (2001) reported greater beta-band activation of the frontotemporal cortex in the right hemisphere of individuals with a choleric (i.e., extraverted) temperament and greater activation in the left hemisphere for persons with a melancholic (i.e., introverted) temperament. EEG oscillation in the alpha band frequency (8–13 Hz) is also modulated by the tempo transformations of a musical piece (Ma et al., 2012). In addition, EEG oscillations in the theta band (the 4–8 Hz) increase with cognitive demands during verbal and spatial working memory (Krause et al., 2000; Tesche & Karhu, 2000) and episodic memory retrieval (McNamara & Ballard, 1999; Sederberg et al., 2003). The theta band power is also a marker of increased arousal or attention (Barry et al., 2009; Faller et al., 2019). Because of the differences inherent in the various frequency bands, topography and reactivity to specific tasks (Kropotov, 2016), as well as functional differences (Moretti et al., 2013), it may be insensitive to the detection of relevant neural processes if EEG is analyzed by the general range (Egner & Gruzelier, 2004; Gruzelier, 2014; Mirifar et al., 2019; Pimenta et al., 2018). Thus, researchers often subdivide cortical activity into some minor frequencies. For example, alpha and beta EEG oscillation bands can be further separated into low-alpha (8–10 Hz), high-alpha (11–13 Hz), low-beta (13–20 Hz) and high-beta (21–30 Hz) power, that collectively serve to capture cerebral cortical activity in a comprehensive manner (Mirifar et al., 2019; Pimenta et al., 2018). As such, it is important to examine all frequency bands when assessing cortical arousal during engagement with a task.

The experimental paradigms typically employed in previous studies required participants to passively listen to music and used genre-based methods of self-reporting (Delsing et al., 2008; Rentfrow, Goldberg & Levitin, 2011). In the present study, we examined tempo preference and cortical activity by employment of a freely chosen musical recall task during which participants tapped on a keyboard as we recorded their EEG activity. Such an approach was deemed more likely to reveal a native response. The overarching goal of the present study was to assess a motivational basis for choosing to become a trained ballroom dancer, which as this is an important element in the motivational basis of human performance and sport performance. The specific aims of the study were (1) to determine if the sensory stimulation (i.e., tempo) of preferred or freely chosen music was higher in dancers than controls; (2) to assess if such a population was characterized by sensation-seeking (i.e., consistent with the gravitational hypothesis); and (3) to assess the magnitude of cerebral cortical activation during preferred melodic recall. We hypothesized that the preferred tempo for dancers would be faster than that for nondancers and that this preference would covary with the presence of an extraverted temperament. We further hypothesized that EEG beta power would be elevated in dancers relative to controls during freely chosen melodic recall and, finally, that tempo preferences are positively related to excitatory neural activity (i.e., EEG low- and high-beta power) in this population.

## Materials and Methods

### Participants

To achieve a statistical power of 80% to conduct statistical assessments of the behavioral measures, a power analysis indicated that 128 participants were required. Seventy ballroom dancers (aged 20.09 ± 1.40 years; 28 males) and 71 nondancers (aged 22.59 ± 2.17 years; 26 males), all of whom were of Chinese nationality, were recruited from flyers posted in the student lounge describing the study and asking for volunteers. All of the dancers majored in dance sports, had at least 5 years of experience and were right-handed. They had no observable cognitive dysfunction, motor or mental disorder that could affect their ability to perform the task.

To achieve a statistical power of 80% to conduct statistical assessments of the psychophysiological measure, a power analysis indicated that 40 participants were required. A subset group of 29 dancers (aged 19.81 ± 1.82 years; 10 males) and 30 nondancers (aged 23.06 ± 2.52 years; 10 males) also were tested with EEG to explore the neural mechanisms of preferred tempo while they completed the tapping task. The study procedures were consistent with the ethical standards of the Research Ethics Committee and with the 1964 Declaration of Helsinki. The experiment was approved by the ethics committee of the Shanghai University of Sport (No. 2015003) and all participants read and signed an informed consent form before the experiment. Participants were compensated for their participation.

### Temperament measure

The Chen Huichang 60 Temperaments Inventory is a self-report questionnaire that uses a 5-point Likert scale with 60 items scored from −2 (very inconsistent) to 2 (very consistent), having 15 items associated with each temperament type. For instance, “*I’d rather work alone than have a lot of people together* (*宁可一个人做事，不愿与很多人在一起*)”, “*I am good at interacting with people* (*善于和人交往*)”, “*Act rashly, often without thinking of the consequences* (*做事有些莽撞，常常不考虑后果*)” (Chen, 1995). The four temperament types (sanguine, choleric, phlegmatic, and melancholic) were identified separately, and the total score was obtained for each type. If the score of a certain temperament is more than four points higher than the others, it is regarded as this type of temperament. Furthermore, if the scores of the varying types are very close and higher than the other temperament, the subject is considered to belong to a mixture of these temperaments. For example, if the difference in score between the two temperaments is not more than three points, but more than four points higher than the other two temperaments, the subject is also considered to belong to the mixed type of two temperaments. The reliability coefficient of the scale was 0.86, and the criterion validity was 0.68. Internal consistency was acceptable, with Cronbach’s α of 0.79.

### Procedure

To achieve the purpose, we assessed temperament and preferred tapping tempo during melodic recall in dancers and controls during an initial phase of data collection. To further explore the neural activation, we ran a second phase of data collection, which was identical to the first phase in terms of the temperament assessment, however, EEG was also collected during melodic recall and execution of tapping. As such, we examined how temperament and cerebral cortical activation were related to tempo preference from both behavioral and neural levels of measurement through employment of the second phase of testing.

Participants first sat alone in a quiet room and completed a temperament questionnaire. Second, they performed a tapping task for which they were asked to recall their favorite music piece and tap to the tempo using the letter “M” on a computer keyboard for 40 taps. A computer (architecture, X64; system Windows 7 Ultimate) provided visual instructions and recorded tapping data using routines from Psychtoolbox 3.0 running on MATLAB 2013a (The MathWorks, Inc.). The screen (pixel resolution, 1,440 × 900; frame rate, 60 Hz; size, 19.5 inches) was placed 80 cm in front of the seated participants. In addition, we also recorded the EEG (to assess theta, alpha, and beta band power) across the scalp topography during the recall period in subset group, which was the time taken to complete the 40 taps in the subsample of participants.

### EEG recording and data processing

The EEG was recorded using 64 Ag/AgCl electrodes placed on the scalp according to the international 10–20 system. The ground was placed at AFz and was referenced to the FCz site on-line. Re-referencing was achieved with two electrodes placed on the mastoids in pre-processing. The horizontal electrooculogram was recorded at the outer canthus of the right eye, and the vertical electrooculogram was recorded below the left eye. Impedances of all electrodes were kept under 5 kΩ. EEG was recorded continuously until the participant finished tapping and was digitized at a sampling rate of 1000 Hz (BrainAmp Standard amplifier, Brain Products GmbH, Munich, Germany).

EEG preprocessing was performed with the software Brain Vision Analyzer 2.01 (Brain Products GmbH, Munich, Germany). The EEG data were then analyzed offline using EEGLAB (Delorme & Makeig, 2004) in Matlab 2013a. The preprocessing consisted of rejecting artifacts in the time and frequency domains. Raw data were visually inspected by an experienced data analyzer to remove major artifacts caused by body movements. Channels with excessive artifacts were interpolated by the four nearby good-quality channels. Basic filters were applied in the following order: a 50-Hz notch, a 1-Hz high-pass and a 30-Hz low-pass filter. Furthermore, independent component analysis (ICA) was applied to the preprocessed data using the InfomaxICA algorithm (Lee, Girolami & Sejnowski, 1999). The data were spatially filtered by ICA to remove blink and eye movement artifacts. EEG data from free tapping was extracted after ICA, which was time-locked to the onset of the first tapping, and included a pre-tapping period of 2 s (baseline). The baseline was then subtracted from the epoched data.

Analysis of the power spectrum was conducted by performing short-time Fourier transform (STFT) (Auger & Hlawatsch, 2008) on the free-tapping EEG data. The STFT was conducted with the following basic parameters: a 1-s time window, 500 overlapped samples, and 5000 discrete Fourier transform points. The power spectrum was calculated using a periodogram estimate.

### Statistical analysis

The preferred music tempo was calculated as beats per minute (bpm) to represent the rate. The standard deviation (SD) of the beat among the 40 repeated taps was also examined to evaluate the variability of an individual’s preferred tempo. Independent *t*-tests were conducted to determine if there was a difference in the preferred tempo and variability between the two groups. Multivariate analysis of variance (MANOVA) and a 2 (Group: dancer vs. nondancer) × 4 (Temperament: choleric, sanguine, phlegmatic, and melancholic) ANOVA was applied to the scores of the four temperament types to determine differences between and within the groups. A series of correlational analyses was also conducted to assess the association between preferred tempi and temperament in each group via the Pearson correlation coefficient.

For EEG data, a 2 (Group: dancer vs. nondancer) × 2 (Hemisphere: left vs. right) × 5 (Region: frontal, central, parietal, temporal, and occipital regions) ANOVA (i.e., electrodes F3, F4, C3, C4, P3, P4, T7, T8, O1 and O2 were selected to represent homologous sites within each brain region) and a separate 2 (Group: dancer vs. nondancer) × 4 (Midline electrode location: Fz, Cz, Pz and Oz) ANOVA were applied to examine theta (4–8 Hz), low-alpha (8–10 Hz), high-alpha (11–13 Hz), low-beta (13–20 Hz) and high-beta (21–30 Hz) power. Correlation (i.e., Pearson coefficient) analyses were also conducted to examine any associations between preferred tempo and brain dynamics (i.e., EEG power).

## Results

### Preferred tempo

Independent *t*-tests applied to the preferred tempo revealed that the tempo preferred by dancers (159 ± 65 bpm) was significantly faster than that preferred by nondancers (132 ± 66 bpm), *t* = −2.427, *p* = 0.016, Cohen’s *d* = 0.409, but there was no significant effect for SD, ps > 0.05.

### Temperament type

**Figure 1:**
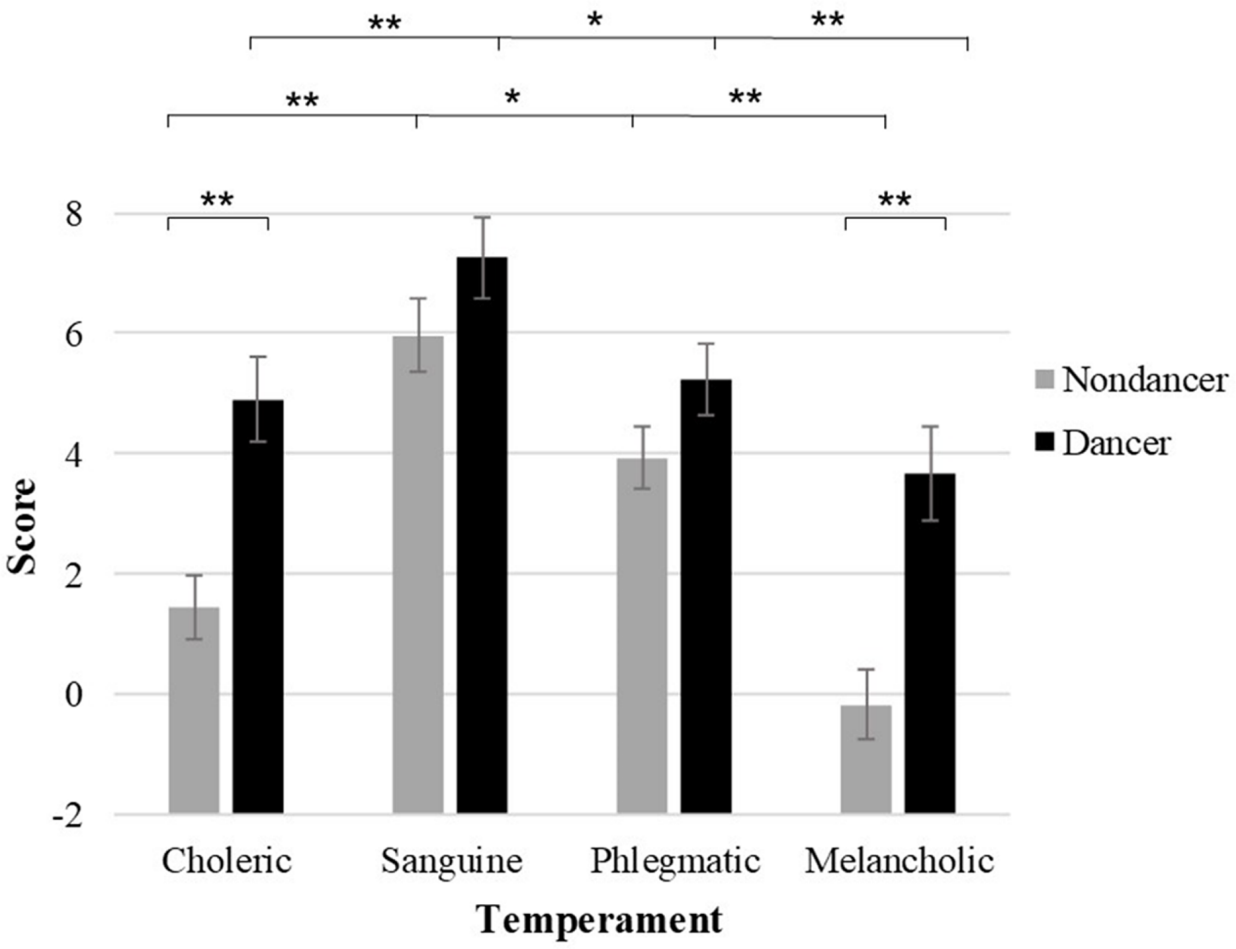
Temperament traits. Asterisks represent statistically significant differences in the scores between and within the two groups. Error bars represent standard error; *p < 0.05, **p < 0.01 for the indicated comparisons.

The MANOVA applied to the four temperaments revealed that choleric and melancholic scores in dancers were higher than those in nondancers, *F*_(1,141)_ =15.730, *p* < 0.0001, η_p_^2^ = 0.102 and *F*_(1,141)_ = 15.723, *p* < 0.0001, η_p_^2^ = 0.102, respectively. In addition, the 2 × 4 ANOVA revealed a significant interaction between Group and Temperament, *F*_(3,417)_ = 3.994, *p* = 0.013, η_p_^2^ = 0.028. A simple effects analysis indicated that the sanguine temperament was significantly higher than the scores for the other three temperaments within the dancers and these (i.e., choleric, phlegmatic and melancholic) are illustrated with designation of any differences in Fig. 1. Nondancers also showed the sanguine temperament was significantly higher than the other temperaments exhibited by that group (ps < 0.05), and that the phlegmatic temperament was also higher than the choleric and melancholic temperaments, both values of *p* < 0.05 (Fig. 1).

### EEG theta power

The 2 (Group: dancer vs. nondancer) × 2 (Hemisphere: left vs. right) × 5 (Region: frontal, central, temporal, parietal and occipital lobes) ANOVA revealed a significant effect of Region, *F*_(4,590)_ = 54.053, *p* < 0.001, η_p_^2^ = 0.275. Specifically, the frontal lobe exhibited greater power relative to the other lobes in all study participants, all ps < 0.001, and that the central region exhibited greater power than the parietal, temporal and occipital lobes, ps < 0.01.

The 2 (Group: dancer vs. nondancer) × 4 (Midline electrode location: Fz, Cz, Pz and Oz) ANOVA revealed a significant effect of Midline location, *F*_(3,236)_ = 30.920, *p* < 0.001, η_p_^2^ = 0.289, such that Fz exhibited the greatest theta power and Oz exhibited the smallest, ps < 0.05.

### EEG low-alpha power

The 2 × 2 × 5 ANOVA revealed a significant main effect of Region, *F*_(4,590)_ = 6.489, *p* < 0.001, η_p_^2^ = 0.044, such that the temporal lobes exhibited lower low-alpha power than that exhibited in the frontal, parietal and occipital lobes, ps < 0.05.

The 2 × 4 ANOVA revealed no effect of Midline location, *F*_(3,236)_ = 0.141, *p* = 0.935, group, *F*_(1,236)_ = 1.327, *p* = 0.251, or any interaction between Group and Midline location, *F*_(3,236)_ = 0.146, *p* = 0.932.

### EEG high-alpha power

The 2 × 2 × 5 ANOVA revealed a significant effect of Region, *F*_(4,590)_ = 8.092, *p* < 0.001, η_p_^2^ = 0.054, such that the occipital lobe exhibited greater power than that observed in the frontal, central and temporal lobes, ps < 0.01, while the parietal lobe exhibited greater power than that in the temporal lobe, *p* = 0.019.

The 2 × 4 ANOVA revealed no effect of Midline location, *F*_(3,236)_ = 0.863, *p* = 0.461, group, *F*_(1,236)_ = 0.001, *p* = 0.981, or interaction between Group and Midline location, *F*_(3,236)_ = 0.244, *p* = 0.866.

### EEG low-beta power

**Figure 2:**
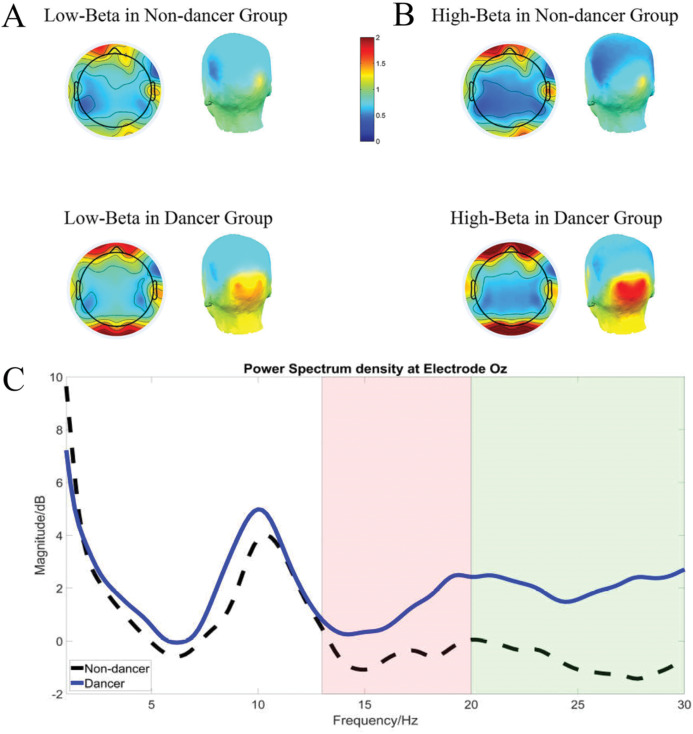
Average power spectra of low-beta and high-beta power in dancer and nondancer groups. Topographic and 3-dimensional images of the power spectrum density in the low-beta band (A) and the high-beta band (B) in the nondancer and dancer groups. (C) Overall power spectra density in nondancers and dancers at electrode Oz.

The 2 (Group) × 2 (Hemisphere) × 5 (Region) ANOVA revealed that the effect of Group was significant, *F*_(1,590)_ = 4.05, *p* = 0.045, η_p_^2^ = 0.007, such that dancers exhibited higher low-beta power than nondancers. Additionally, the 2 (Group) × 4 (Midline location) ANOVA also revealed an effect of Group, *F*_(1,236)_ = 3.876, *p* = 0.050, η_p_^2^ = 0.017, such that dancers exhibited greater power than nondancers (Fig. 2A).

### EEG high-beta power

**Figure 3:**
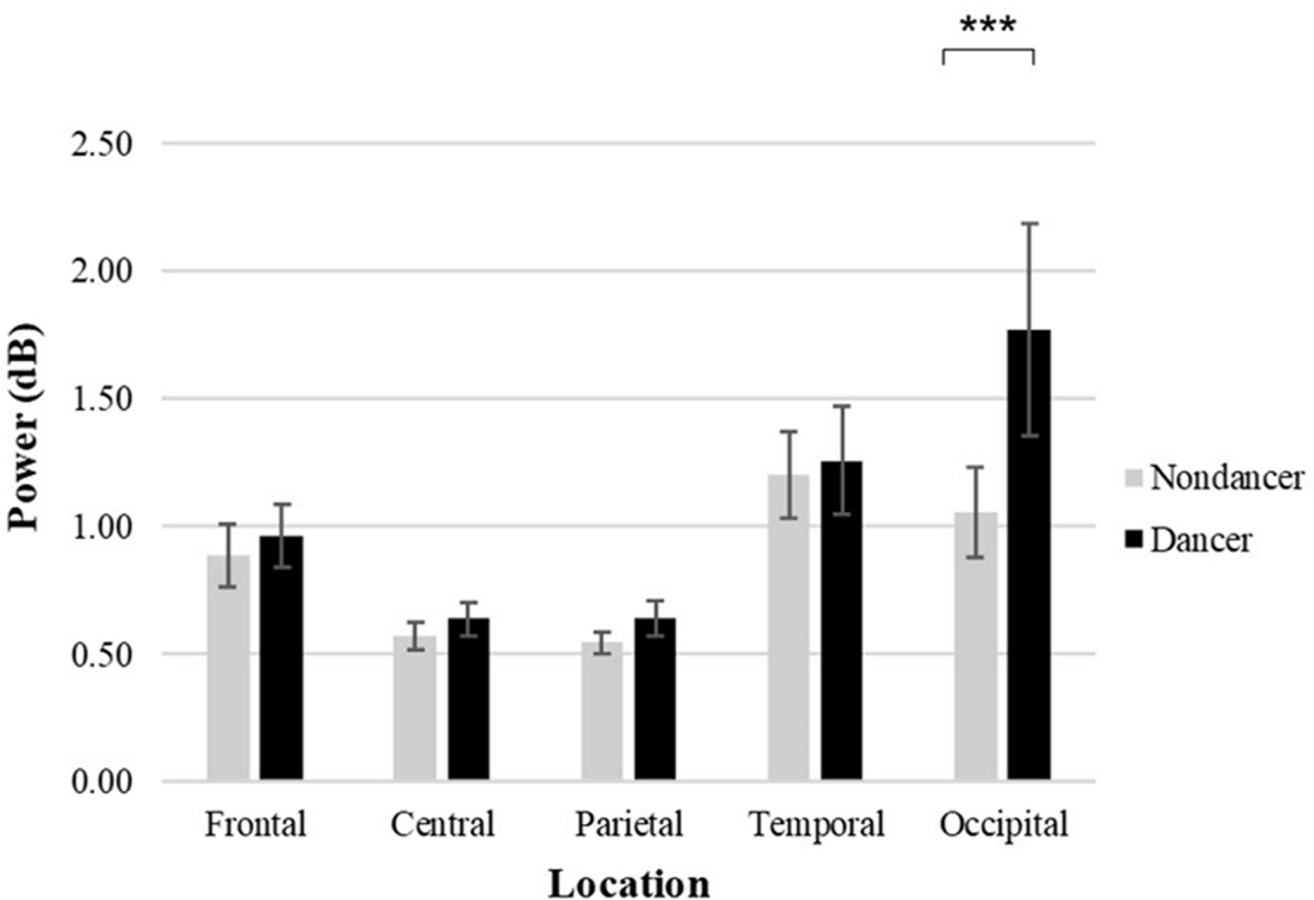
High-beta power at five locations in the brain for the two groups. Error bars represent standard error; ***p < 0.001 for the indicated comparison.

The 2 (Group: dancer vs. nondancer) × 2 (Hemisphere: left vs. right) × 5 (Region: frontal, central, temporal, parietal, and occipital lobes) ANOVA revealed a main effect of Group, *F*_(1,590)_ = 7.091, *p* = 0.008, η_p_^2^ = 0.012, such that dancers exhibited greater high-beta power than nondancers. An interaction between Group and Region was also significant, *F*_(4,590)_ = 2.619, *p* = 0.034, η_p_^2^ = 0.018. A simple effects analysis revealed that the dancers revealed greater magnitude of difference in power in the occipital lobe than nondancers did (*p* < 0.001). In addition, within the dancer group, the occipital lobe exhibited greater power than the frontal, central, parietal (all values of *p* < 0.001) and temporal regions (*p* = 0.048); and the temporal lobe revealed greater high-beta power than the central and parietal regions, values of *p* = 0.007 (Fig. 3).

The 2 (Group: dancer vs. nondancer) × 4 (Midline electrode location: Fz, Cz, Pz and Oz) ANOVA also indicated that the effect of Group was significant, *F*_(1,236)_ = 7.028, *p* = 0.009, η_p_^2^ = 0.030, such that dancers exhibited greater high-beta power than nondancers. The interaction between Group and Midline location was also significant, *F*_(3,236)_ = 3.089, p = 0.028, η_p_^2^ = 0.039. The simple effects analysis revealed that dancers exhibited greater power in the occipital lobe than nondancers (*p* < 0.001). Finally, dancers exhibited greater high-beta power in the occipital than the central, frontal, and parietal lobes, all values of *p* < 0.001 (Figs. 2B and 2C).

### Correlational analyses

Within dancers, the preferred tempo was negatively correlated with high-beta power at midline electrode sites Fz (*r* = −0.447, *p* = 0.015), Cz (*r* = −0.406, *p* = 0.029), Pz (*r* = −0.446, *p* = 0.015) and Oz (*r* = −0.392, *p* = 0.035). Such a directional relationship was also found within dancers for low-beta power at the Oz electrode site (*r* = −0.372, *p* = 0.047).

## Discussion

Given that individuals differ in the activities they pursue, the present study explored the role of temperament, as related to the need for stimulation, and cerebral cortical activation in a population engaged in high-tempo human movement performance. Specifically, this aim was addressed by examining these variables in individuals with a history of engagement with arousing stimuli (i.e., fast musical tempo in ballroom dancers). Dance is a type of movement embraced by individuals to express emotion or affect; however, the musical tempo preference and temperament traits of dancers have received little attention in the literature. Therefore, the present study addressed this gap and, furthermore, subscribed to a multilevel analysis by additional consideration of psychophysiological assessment of neural activity during melodic recall. Relative to nondancers, we observed an extraverted temperament (i.e., need for heightened arousal) in ballroom dancers accompanied by a preference for a faster musical tempo. This was complemented by the finding of elevated EEG beta activity, indicative of heightened cerebral cortical arousal, during their melodic recall. These results are consistent with arousal theory (Eysenck & Eysenck, 1985), suggesting that such dancers seek elevated musical stimulation relative to nondancers, thus providing for heightened cortical activation, which was detected. The present results help to explain the behavioral choices that ballroom dancers make for engagement with energetic music and are suggestive of a gravitational hypothesis to explain the association with inner arousal requirement and environment stimuli relevant to this specific form of cognitive-motor activity (Eagleton, McKelvie & De Man, 2007).

Although the dancers showed a temperamental profile supportive of extraversion they also demonstrated, paradoxically, an element of heightened introversion (i.e., melancholia) compared to nondancers, which appears contradictory. Such a conundrum may be resolved by consideration of interactional personality theory (Endler & Magnusson, 1976), which explains the individual’s behavior by a continuous and multidirectional interaction between person and situational variables that they encounter. In addition, cognitive factors, emotional factors and the psychological meaning or perception of the situation play vital roles (Endler & Magnusson, 1976). In essence, the personality dimension of extroversion is relevant to the perception of the arousing aspect of musical stimuli while the trait introversion was not relevant to the perception. As such, the perception is critical as to the influence of latent disposition. While they tapped during melodic recall, the dancing scene they encountered might be recurred, influenced their tapping behavior. In addition, dancers, in a way, are drawn to perform extremes of emotion rather than bland middle range, thus, other kind of ballroom dancing (e.g., tango) would seem to represent less of sensation seeking and more of subdued moods.

As shown in Fig. 1, all four types temperament in dancer group are higher than nondancers, suggesting that dancers are potentially more variable in their reactivity depending on the perception of a situation. Specifically, the temperament assessment revealed different temperamental patterns between the groups, showing both elevated choleric as well as elevated melancholic temperaments in dancers relative to the nondancers. A choleric temperament literally means “yellow bile,” and indicates that an individual with this temperament tends to be energetic, ambitious, irritable and furious. Thus, people with this temperament would reasonably prefer a faster tempo in steps, paces, and music, which was consistent with the finding of a faster beat preference during the tapping task. Within-group comparisons also revealed that the sanguine temperament (stable extraverts) was the highest of the four traits in the dancers. Again, this finding is consistent with the gravitational hypothesis of pre-existing differences, which serve as predispositions to behavior (Eagleton, McKelvie & De Man, 2007). In other words, our traits drive us towards certain behaviors like the seeking of arousal (i.e., fast-tempo dance). But the observation of elevated melancholia initially appears puzzling. As a tenable resolution of the apparent contradiction, it is reasonable that ballroom dancers perceive the performance environment as one that provides an opportunity for sensory stimulation, which is a situationally specific perception and one that is relevant to the dispositional tendency of an extraverted personality type. Consistent with the framework of interactional personality theory, it is also tenable that the introversion trait (i.e., melancholia) would be irrelevant to the dancer’s perception of the situation (i.e., one that is arousing) thus rendering the trait impotent in its influence on behavior. Some have theorized that temperament traits, considered in isolation of the perception of a situation, are limited or weak in terms of accounting for explained variance in behavior. However, consideration of situational perception, in tandem with consideration of a relevant underlying personality trait, can explain observed behavior and the motivation to perform a given task or challenge (Endler & Magnusson, 1976). In this manner, dispositional extraversion would facilitate engagement in motor activities to which an individual is exposed and perceived as arousing, thus becoming appetitive. This explanation is reasonable in the present sample given the characteristics of ballroom dance. Although no correlations were observed between preferred tempo and temperament in either group, such absence may also be due to the limited power of personality traits, by themselves without consideration of situational perception, to explain specific behaviors (Morgan et al., 1988). That is, personality traits are necessary, but not sufficient variables, to understand why individuals engage in particular types of human performance.

The finding of a higher tempo preference in dancers vs. nondancers also supports those of previous studies of the perception of dance music (Moelants, 2003, 2008). Ballroom dancing, especially competitive international Latin dancing, is more energetic than some other types of dancing. The couples perform dazzling footwork and provocative movements coordinated with either enthusiastic or soothing music. Dancesport is another name for competitive ballroom dancing, adding the element of sports and reaching for a high standard in competitive movements. Ballroom dancers practice their skills regularly by tapping to every beat on time with various types of dance music. Given the theory of resonance (Large, 2008) beyond the conceptual frameworks outlined above, it is not surprising that ballroom dancers preferred a faster and stimulating tempo during melodic recall.

In support of the arguments above, Rentfrow & Gosling (2003) also reported that upbeat and conventional music is positively correlated with extraversion, agreeableness, and conscientiousness, whereas it is negatively associated with openness. Extraversion and agreeableness are also positively associated with the energetic and rhythmic dimensions of music. Consistent with these findings, individuals who are considered extraverted and agreeable prefer pop, soundtrack, funk, electronic, and dance music genres (Greenberg et al., 2015). An online study that accumulated a large sample size to examine whether an association existed between Facebook “Likes” for musical artists and individual differences in personality (Nave et al., 2018) indeed found a relationship between personality traits and music preference, providing support for these associations. Brown (2012) explored correlations with personality and music in Japanese university students and found that openness and “aesthetic appreciation” are associated with reflective music preference (e.g., classical, jazz), and sociability (one facet of extraversion) is correlated with a preference for pop music. Despite such studies of personality and music preference, some results have been inconsistent and effect sizes have been small (Schäfer & Mehlhorn, 2017). This raises the question whether additional factors are needed to explain individual differences within music preference.

Although the associations found between neural activity and personal traits has a long history (Delsing et al., 2008; Geen, 1984; Kantor-martynuska, 2009), the linkage with music preference is relatively unknown. EEG is an effective way to detect cortical neural activation, thus, we also examined neural activity during the tapping task in both groups to examine their inner activation during preferred tempo recall process. Compared with nondancers, both high-beta and low-beta EEG power were higher in dancers, with high-beta power being greatest in the occipital lobe. We recorded the EEG (and extracted and analyzed theta, alpha, and beta power) across the scalp topography during the melodic recall period, defined by the time taken to complete the 40 taps. A previous study reported by Bauer, Kreutz & Herrmann (2015) revealed that the preferred tempo was significantly related to the frequency of the motor beta activity, providing evidence for a specific characteristic of preferred tempo. The present study also revealed a relationship between preferred tempo and EEG beta power in dancers. Oscillations in the beta frequency band have been found in other motor tasks (Neuper & Pfurtscheller, 2001) and have also been shown to be related to anticipatory activity in an auditory beat perception study (Fujioka et al., 2009), indicating that the beta frequency band plays an important role in music perception and motor regulation. It is noteworthy that the dancers showed heightened cortical activity during melodic recall, implying the achievement of neural stimulation that they tend to seek. However, the current study design cannot alternatively determine whether the heightened EEG beta effect seen in the dancers is due to the music recall or the faster tapping, it could be both factors are associated with the EEG for all participants. While recalling preferred tempo, dancers, who are more inclined to experience such body movement, may implicitly recall motor procedures or movements associated with tempo (i.e., simulating a dancing session). Consequently, beta power, in this case, may indicate an inhibition mechanism through which a dancer may keep the attention on the task (Tempel, Frings & Pastötter, 2020). The negative correlation observed between EEG beta power and preferred tempo at Oz within the dancer group may be indicative of a compensatory arousal strategy. More EEG beta power in dancers, slower tempo they preferred. More specifically, dancers who exhibited lower EEG beta power may seek a faster tempo to achieve desired stimulation while those who exhibit heightened arousal moderate the preferred beat of the music to manage arousal. Thus, the present study provides further evidence supporting the importance of the beta frequency band in motor behavior in skilled cognitive-motor behavior. Interestingly, the occipital areas were related to dance observation (Burzynska et al., 2017), as well as movement planning and execution process (Cappadocia et al., 2017), implying more active for dancers than nondancers during preferred tempo recall. It comes to the assumption whether the dancers were imagining themselves dance or gesture, adding evidence to study dance training-induced plasticity in the brain (Burzynska et al., 2017).

Although devoid of main or interactive effects with group membership (i.e., dancers vs. controls), the regional topographical effects noted for EEG theta, low-alpha and high-alpha band power contribute to confidence in the findings based on the distribution of regional activity. For example, a significant main effect of elevated frontal theta, relative to all other regions, was noted during melodic recall. Such an effect implies the engagement of working memory during the tapping task in all the study participants while the regional distribution of elevated high-alpha power in the occipital region is consistent with the typically topography of EEG alpha band power (Bae & Luck, 2018; Tesche & Karhu, 2000).

In summary, an association between personality and music preference was previously reported by several groups (Brown, 2012; Chamorro-Premuzic, Fagan & Furnham, 2010; Delsing et al., 2008; Greenberg et al., 2016; Rentfrow & Gosling, 2003). Accordingly, we examined the usefulness of a theoretical perspective relevant to sensation seeking and arousal and employed a validated trait to further explore the association of temperaments with behavior in dancers. The present results revealed that both dancers and nondancers exhibited evidence of extraversion. Although choleric and melancholic temperaments received higher scores among dancers than among nondancers, the sanguine temperament (stable extraverts) received the highest score within the dancers. Based on the arousal theory, extraverts typically exhibit a lower level of spontaneous cortical arousal (Razoumnikova, 2003), contributing to their preference for upbeat and energetic music (with a faster tempo) to achieve a comfortable level of arousal. Ballroom dancing is a competitive sports event characterized by emotional investment and motivated behavior. Persons with higher choleric temperament scores, as observed in the dancers, are considered neurotic extraverts, with less arousal in the central nervous system than introverts, and thus need elevated environmental stimuli to activate the brain in a compensatory manner, which may underlie their preference for an upbeat tempo.

The present experimental paradigm employed a new tapping task, one that allowed participants to freely recall their preferred tempo and tap that tempo on a keyboard. This method differed from the category-based and self-report methods used in previous publications (Brown, 2012; Chamorro-Premuzic, Fagan & Furnham, 2010; Delsing et al., 2008; Greenberg et al., 2016; Rentfrow & Gosling, 2003). Our intention was to explore tempo preference using a more specific and accurate method that enabled a view of the native tendency. The significant result of a faster tempo preference among dancers suggests that they may be influenced via experience by their synchronized music experience, but also by their dispositional tendencies. We have empirical evidence of the latter, but cannot dismiss the former. Beyond arousal theory and the gravitational hypothesis, the present results also supported the resonance theory (Large & Kolen, 1994; Large, 2008; Van Noorden & Moelants, 1999), such that dancers keep pace with dance music, synchronizing their movements and neural activity with the tempo and thus contribute to the creation of resonance for various types of dance music at preferred tempi.

## Conclusions

The present results are consistent with arousal theory, suggesting that dancers need more stimulation and choose active and energetic music (faster tempo), providing the nervous system with higher arousal levels. To our knowledge, the present study is the first to use a modified voluntarily tapping task to examine tempo preference in a natural manner. Moreover, the study focused on a specific population within the performing arts and subscribed to a multilevel approach consisting of measures of temperament and psychophysiology, thus providing an integrated perspective of the tempo preference of dancers. The finding that temperament was associated with heightened brain activity during musical recall raises the possibility of a mechanistic relationship that offers a promising line of investigation for future studies. A fundamental question is whether the observed preference in tempo is learned as a result of practice and experience or, conversely, whether a dancer’s dispositional tendencies contribute primarily to tempo preference. In essence, is it learning or gravitation driven by personality that primarily drives the engagement to achieve skill and high levels of human performance? This is an age-old question, but the present investigation supports a role, whether primary or not, of temperament in human performance.

## Supplemental Information

10.7717/peerj.10658/supp-1 Supplemental Information 1 Behavior & temperament raw data. Each data point indicates the tempo preference and score of temperament of each participants.

10.7717/peerj.10658/supp-2 Supplemental Information 2 EEG data. Each data point indicates the power of theta, low alpha, high alpha, low beta and high beta in each region and hemisphere for each participants.

10.7717/peerj.10658/supp-3 Supplemental Information 3 Raw data codebook. Each data point indicates the code in raw dataset file and their paraphrase.

## Additional Information and Declarations

### Data Availability

The following information was supplied regarding data availability: Raw data, including the temperament score, tapping tempo, and power of cerebral cortical activity for all participants, are available in the Supplemental Files.
